# HPV vaccines for circumpolar health: summary of plenary session, “Opportunities for Prevention: Global HPV Vaccine” and “Human Papillomavirus Prevention: The Nordic Experience”

**DOI:** 10.3402/ijch.v72i0.21070

**Published:** 2013-08-05

**Authors:** Eileen F. Dunne, Anders Koch

**Affiliations:** 1 Division of STD Prevention, National Center for HIV/AIDS, Viral Hepatitis, STD and TB Prevention, Atlanta, GA, USA; 2 Department of Epidemiology Research, Statens Serum Institut, Copenhagen, Denmark

**Keywords:** HPV vaccine, Nordic countries, circumpolar health, HPV

## Abstract

In this publication, we provide an overview of the presentations, “Opportunities for Prevention: Global HPV Vaccine” and “Human Papillomavirus Prevention: The Nordic Experience” as a part of the 15th International Congress on Circumpolar Health, held at Anchorage, Alaska, on August 8, 2012. We provide an overview of HPV, HPV vaccines and policy as well as the Nordic experience with HPV vaccine introduction.

Human papillomavirus (HPV) is a common infection and is species specific. There are more than 100 HPV types, 40 of which infect the mucosa, including the genital mucosa. The virus is composed of outer capsid proteins (L1 and L2), enclosing a small 8-kilobyte double-stranded, circular DNA. The capsid proteins will self-assemble into empty capsid shells when produced in a cell culture. These empty capsid shells, or virus-like particles (VLPs), resemble a wild-type virus but contain no infectious material. This unique property of the capsid proteins was essential for vaccine development as the proteins provide an antigen with conformational epitopes. Animal models were fortunately available to facilitate vaccine development. Production of bovine papillomavirus VLPs and cottontail rabbit papillomavirus VLPs demonstrated that the response to these proteins prevented infection and disease in these animals (1).

HPV infection results in a variety of diseases and cancers. Low-risk or non-oncogenic HPV cause genital warts and a condition called recurrent respiratory papillomatosis (RRP), in which warts grow in the throat and/or respiratory tract. High-risk or oncogenic HPV cause various cancers and precancers. Oncogenic HPV types cause virtually all cervical cancers (CC), and subset of vaginal, vulvar, anal, penile and oropharyngeal cancers (2). For most of these cancers, there are no prevention strategies. Secondary prevention for CC includes CC screening with the Pap test or other methods. CC prevalence has decreased in industrialized countries over decades, but in many parts of the world, including Africa, India and South America, it is still a substantial problem. Worldwide, the ability to implement comprehensive CC screening is limited due to resource and infrastructure needs for this programme. More than half a million CC cases, and a quarter million deaths due to CC, occur each year globally (3).

## HPV vaccines

There are 2 prophylactic HPV vaccines available, and these vaccines have similarities and differences. The 2 vaccines are bivalent vaccine (Cervarix) and quadrivalent vaccine (Gardasil) (Table I). Both vaccines are composed of VLPs, but the vaccines have differences in how the VLPs are manufactured, which VLPs are included and the adjuvants. Gardasil prevents HPV types 16, 18, 6 and 11 infection (2 HPV types that cause CC and 2 others that prevent genital warts) and Cervarix prevents HPV types 16, 18 infection (2 HPV types that cause CC).

**Table I:** HPV vaccines

	Quadrivalent (Gardasil)	Bivalent (Cervarix)
Manufacturer	Merck	GlaxoSmithKline
VLP types	6, 11, 16 and 18	16 and 18
Dose of protein	20/40/40/20 µg	20/20 µg
Producer cells	*Saccharomyces cerevisiae* (yeast)	*Baculovirus-infected Trichoplusiani* insect cell line
Adjuvant	AAHS:	AS04:
	225 µg amorphous aluminum hydroxyphosphate sulphate	500 µg aluminum hydroxide 50 µg 3-*O*-desacyl-4′-monophosphoryl lipid A
Schedule (IM)	3 dose series	3 dose series

Abbreviations: VLP, virus-like particle; IM, intramuscular.

Both vaccines have been shown in clinical trials to have high efficacy (>95%) for prevention of cervical precancers (4, 5). Gardasil has been found to have high efficacy for the prevention of genital warts, anal precancers, and vulvar/vaginal precancers (4, 6). In the United States, both Cervarix and Gardasil are recommended for females and Gardasil is recommended for males. The duration of antibody response and efficacy is being followed for both vaccines, but to date there is no evidence of waning of immune response or efficacy for 6–10 years. The vaccines are very safe and were studied in >50,000 persons globally pre-licensure. As of June 2012, more than 46 million doses of quadrivalent vaccine have been distributed in the United States. The main adverse reactions of vaccination have included pain, swelling and redness at the injection site.

## HPV vaccine implementation

In 2009, WHO published a position paper on the recommendations for HPV vaccine worldwide and suggested the most appropriate target group (9- to 13-year-old girls), that HPV vaccine should not divert resources from effective CC screening programmes, and HPV vaccine should be introduced as part of a coordinated strategy for CC prevention (7). HPV vaccine has been primarily implemented in settings such as Western Europe, U.S., Canada, and Australia, but in December 2011, GAVI Alliance (Global Alliance for Vaccines and Immunisation) opened the window for funding which will make way for increased implementation worldwide (8). The revolving fund has included the HPV vaccine that will facilitate implementation in Pan American Health Organization (PAHO) countries (9).

Areas in the circumpolar region implementing vaccine include U.S. (Alaska), Canada, Russia and Nordic countries. These countries and regions vary as far as infrastructure and support for vaccination are concerned, and whether vaccine is included in the public programme. These settings all have indigenous populations with important challenges to health care access, including rural location and language barriers. Alaska has an HPV vaccine programme similar to other parts of the United States, and has specific evaluations in place to monitor vaccine impact through collaborations with South Central Foundation and the Artic Investigations Program (AIP), Centers for Disease Control. These activities include monitoring long-term immunogenicity, evaluating HPV types in cancers from Alaska Natives and evaluating the impact of vaccination on HPV types. In Canada, HPV vaccine has been introduced and recommendations vary by province. Activities in Canada that will be unique and important to worldwide implementation include evaluations of less than 3 dose vaccine efficacy and immunogenicity.

## The Nordic experience

Among the 6 Nordic countries (Denmark, Sweden, Norway, Finland, Iceland and Greenland), 5 have implemented HPV vaccine. However, the primary strategies have varied across these settings. We describe the Nordic experience based on a recent publication reviewing HPV vaccine programmes in the region (10). The Nordic countries are characterized by being relatively wealthy, having mainly publicly financed health systems, with high coverage of vaccination. In addition, the Nordic countries have an effective CC screening programme, which has resulted in decreases in the incidence of CC. Despite an effective screening programme, there is an important burden of CC in these settings. Rates of CC vary significantly among the Nordic countries with Greenland having the highest incidence (25 per 100,000, age-standardized rate by World Standard Population (ASW)) (10) followed by Denmark, Norway, Iceland, Sweden and Finland (4 per 100,000 ASW) (11). Compared with other European countries, Denmark has higher rates of CC, and Finland has lower rates.

Denmark was the first Nordic country to introduce HPV vaccine into the standard childhood vaccination programme, offering quadrivalent vaccine (Gardasil) since January 2009 for girls aged 12 years with a catch-up programme for girls aged 13–15 years. In support of this programme was a health technology report from 2007 that showed that a rapid decrease in HPV 16 and 18 infections could be expected after such a programme. In focus group interviews, parents and young people had positive attitudes towards HPV vaccination, with the cancer vaccine prevention aspect being important, whereas the risk of sexual disinhibition was not reported as being important. There was concern of inequality of health given the high cost of vaccine if not included in a free public vaccination programme. Vaccination of boys was demonstrated to double the expenses but have only a moderate effect on the incidence of CC due to herd immunity. In Denmark, there was strong public support to introduce the HPV vaccine, in particular from organizations such as the Danish Cancer Society. This resulted in the decision to also include older women born in the years 1985–1992 in the catch-up programme in 2012.

In general, Greenland follows a similar programme to the Danish childhood vaccination programme, but the Greenland HPV vaccination programme started later. In addition to having the highest incidence of CC among the Nordic countries, Greenland has a high incidence of sexually transmitted diseases. As of 2007, vaccination with the quadrivalent vaccine (Gardasil) for both genders was planned, but a re-evaluation in 2008 changed the decision to include only girls; the programme that was initiated in 2008 was directed at girls aged 12 years with a catch-up vaccination for girls aged 13–15 years. There was no substantial public debate on HPV vaccination.

In Norway, the HPV vaccination programme was recommended in 2007 and introduced in 2009 with quadrivalent vaccine (Gardasil) given to girls aged 11–12 years. A catch-up programme was considered but not decided upon. In contrast to Denmark and Greenland, there was some media concern about vaccine safety and even allegations of corruption against the Norwegian Cancer Registry and the expert group advising the Norwegian Public Health Institute. There were public concerns about inequality of access to vaccination, if this vaccine was not included in the public programme. The Norwegian Gynecological Society advocated in favour of the vaccine programme.

In Iceland, a report in favour of public HPV vaccination was published in 2007 and bivalent vaccine (Cervarix) was first introduced in 2010 directed at girls aged 12 or 13 years, and later to girls aged 12 years only. There was no public debate on HPV vaccination, probably as a result of the financial crisis and other competing public priorities.

In Sweden, a consensus conference in 2006, and a cost-effectiveness report in 2008, recommended HPV vaccination to girls aged 10–12 years, as well as a catch-up programme up to age of 18 years. However, the Swedish Council on Health Technology raised concerns of vaccine safety. Despite this, in 2008 a decision was made to introduce vaccine in 2010. The first public tender amounted to 3.5 million Euros for Cervarix and 5.3 million Euros for Gardasil, but protests from gynaecologists against non-protection from genital warts and a legal appeal by Merck on the tender, resulted in a new tender in 2011 favouring Gardasil. This, in contrast, led GSK to make a legal appeal, but by November 2011 a national programme using Gardasil for girls aged 11–12 years was started.

Finland has the lowest rates of CC in Europe, which may have played a role in their considerations of HPV vaccination. In 2008, an advisory committee was established that by 2011 recommended an HPV vaccine programme for girls aged 11 or 12 years as well as a catch-up programme for girls aged 13–15 years. The programme was endorsed in 2011 by the National Institute of Health and Welfare, but a final decision about the programme was postponed until 2014. Factors influencing the decision not to immediately introduce the vaccine, included effectiveness of CC screening, no demonstration of the ability of the vaccine to prevent cancer, lack of Finnish data on vaccine cost-effectiveness as well as burden of HPV infections, and finally, lack of funding. A number of organizations, including the National Council of Women, the Finnish Cancer Society, the Finnish Association of Midwives, and the Finnish Association of Gynecologists recommended the introduction of an HPV vaccine but to date there is no public programme.

A summary of the HPV vaccine programmes in Nordic countries is included in Table II. In Denmark, vaccine coverage rates for girls born between 1993 and 1998 were 86–90% for the first dose of vaccine, similar to measles vaccine coverage rates given at the same age; 76–83% had completed all 3 doses of the vaccine (12). In Norway, vaccination for girls born in 1998 was 79% for vaccine initiation and 70% for all 3 doses (13). For Sweden, vaccine coverage of girls aged 13–17 years until 2011 was 30% (while not in the public programme) (14); a web-based questionnaire in 2012 showed 80–100% initiation of vaccine in more than 80% of schools with inclusion in the public vaccination programme (15). HPV vaccine coverage rates in other European countries are generally lower than Nordic countries: for school-based vaccination in the United Kingdom and Scotland, coverage rates were 76% (16) and 90% (17) for 3 doses of vaccine; for Slovenia 49% (18) and the Netherlands 50% (19).

**Table II:** Summary of HPV vaccine programmes in the Nordic countries

	Start year	Target ages (years)	Administration	Vaccine	Cost through public programme	Vaccine register
Denmark	R: 2009	R: 12	Medical Home	Gardasil	Free	Planned
	C: 2008	C: 13–15 +20–28				
Greenland	R: 2008	R: 12	Mixed	Gardasil	Free	Partly
	C: 2009	C: 13–15				
Norway	R: 2009	R: 12	School	Gardasil	Free	Yes
Iceland	R, C: 2011–2012	R: 12	School	Cervarix	Free	Yes
		C: 13				
Sweden	R: 2011	R: 11–12	School	Gardasil	R: Free	Yes
Finland	Not decided	–	–	–	–	–

R, routine; C, catch up.

*Source*: Reference 10.

Vaccine prices varied substantially between the countries and from first to second tenders in each country. Greenland and Sweden obtained vaccines at lower cost than Denmark, as Denmark was the first country to introduce the vaccine.

In summary, there are numerous lessons learned from HPV vaccine considerations in Nordic countries. In spite of similar culture and health systems, there are marked differences in HPV vaccination strategies, priorities, debate and concerns. Vaccine introduction was generally supported initially in countries with higher CC rates; first introduction in the high-incidence countries of Denmark and Greenland, and still awaiting introduction in the low-incidence country of Finland. Both the quadrivalent vaccine (Gardasil) and the bivalent vaccine (Cervarix) have been used, although use of the quadrivalent vaccine is more common. There are varying vaccine coverage rates from satisfactory in Denmark to less satisfactory in Norway. Purchasing prices have varied substantially between countries and year of purchase, with later tenders being markedly cheaper than first tenders. There were differences in public debate over vaccination, with a lively debate in Denmark and Norway and no debate in Greenland and Iceland. Public voices ranged from request for vaccination in Denmark to concern about vaccine safety in Norway. Health professionals were active in the debate, most in favour, although there was some concern by health professionals in Norway. There are many interested parties including the Danish Cancer Society lobbying for HPV vaccine programmes and mass media; in Denmark HPV vaccination became a political issue in the Parliament. Vaccine manufacturers in particular have been highly active in the debate in these settings, lobbying, making public complaints, and filing lawsuits. Reasons for public support for HPV vaccination in the Nordic countries, include protection against cancer and reduction in social inequality in health with a public programme, potential sexual disinhibition from vaccination has been of little concern. Because of the varied approaches and issues, the experience of Nordic countries may be useful for other jurisdictions considering vaccine implementation.
